# The effect of diet quality and body mass index on depression in older adults: a growth curve analysis

**DOI:** 10.1186/s12877-024-05392-5

**Published:** 2024-10-15

**Authors:** Yun-Lin Wang, Yun-Han Wang, Dara Kiu Yi Leung, Gloria Hoi Yan Wong, Terry Yat Sang Lum

**Affiliations:** 1 Department of Counseling, Clinical and Industrial/Organizational Psychology, Ming Chuan University, Taoyuan City, Taiwan, Republic of China; 2 Department of Psychology, National Taiwan University, Taipei, Taiwan; 3 Department of Social Work and Social Administration, The University of Hong Kong, Hong Kong, Hong Kong SAR, China; 4 School of Psychology and Clinical Language Sciences, University of Reading, Reading, England; 5 Sau Po Centre on Ageing, The University of Hong Kong, Room 534, 5/F, Jockey Club Tower, Centennial Campus, Pokfulam Road, Hong Kong, Hong Kong SAR, China

**Keywords:** Healthy dietary pattern, Body mass index, Depression

## Abstract

**Background:**

Nutrition not only plays an important role in one’s physical health, but also mental health. The causal association between nutrition and mental health remains unclear. While a healthy dietary pattern may protect one against mental illness, it is also possible that poor mental health could lead to unhealthy eating habits or choices. Furthermore, emerging studies suggest that a higher body mass index (BMI) is associated with a lower risk of depressive symptoms in older adults, contrasting the effect observed in other populations. With an ageing population, this study aimed to investigate the long-term impact of a healthy dietary pattern, BMI, and other covariates on depressive symptoms in older adults.

**Methods:**

We conducted a cohort study between 2014 and 2017, with each follow-up assessment being one year apart. A total of 2081 participants above 65 years old (*M* = 79.65, *SD* = 7.94) completed the baseline assessment in 2014, which included basic demographics, self-reported eating habits, depressive symptoms, and the measurement of height and weight. Distance to supermarkets and fast food was calculated based on participants’ residential addresses. Two growth models were performed to assess the trajectory of change in depressive symptoms over time.

**Results:**

Older adults experienced a significant decrease in depressive symptoms over time (intercept = 2.68, *p* < .001; slope = -0.25, *p* < .001). At baseline, a higher diet quality (B = -0.95, *p* < .001), higher BMI (B = -0.09, *p* < .001), younger age (B = 0.40, *p* = .001), being a male (B = 0.76, *p* < .001), and having fewer chronic diseases (B = 0.39, *p* < .001) were associated with lower levels of depressive symptoms. Over time, a higher diet quality (B = 0.14, *p* = .05), higher BMI (B = 0.02, *p* = .04), and fewer chronic diseases (B = -0.08, *p* < .001) predicted lower levels of depressive symptoms over time.

**Conclusions:**

A higher diet quality and higher BMI may serve as protective factors for depressive symptoms in older adults. Potential implications are being discussed.

**Supplementary Information:**

The online version contains supplementary material available at 10.1186/s12877-024-05392-5.

## Background

Nutrition plays a key role in maintaining a healthy body mass index (BMI, 1) and preventing physical conditions such as sarcopenia [2], cardiovascular diseases, and diabetes [3]. Since the 1990s, there have been emerging studies suggesting that dietary patterns and diet quality could play a role in one’s mental health [4, 5]. For example, the consumption of ultra-processed foods [6], preserved vegetables [7], and meat [8] may increase the risk or exacerbate depressive and anxiety symptoms. In contrast, the vegetable-egg-beans-milk eating pattern has been found to predict lower levels of depression in older adults four years later [7].

Maintaining a healthy dietary pattern and healthy BMI in the older adult population may, however, become more challenging as a result of age-related factors. As mobility and functioning decline with age [9], the quality of the local food environment has been suggested to play an important role in older adults’ diet and weight [10]. Increased access to supermarkets and limited access to fast food has been found to improve diet and weight status [11]. Other factors, such as physical decline (e.g., deteriorated oral health), social (e.g., social isolation) and medical factors (e.g., chronic disease, depression, medications), are also risk factors of increased [12] or decreased appetite in older adults [13]. Irrespective of over or under-eating, both pose a risk of malnutrition, which could lead to an unhealthy body weight [14], poorer functional status, and higher morbidity and mortality rates [15].

The relationship between nutrition and mental health is of great interest to researchers [16]. A healthy dietary pattern can be defined as the consumption of fresh fruit and vegetables, nuts, seeds, whole grains, legumes, and fermented foods that are not ultra-processed or refined [5]. The consumption of macronutrients (e.g., carbohydrates, proteins, and fats), vitamins, and minerals is essential for proper brain functioning, which subsequently influences mental health [17]. As such, having a healthy dietary pattern, in general, is associated with greater emotional well-being and quality of life in older adults [18, 19]. However, research has suggested the possibility of a reverse causal relationship, where mental state influences dietary choices [20]. For example, older adults with depression reported lower intake of fruit and vegetables [21, 22]. Furthermore, in a sample of obese African American, it was found that depressive symptoms were positively associated with the consumption of sugar and fat [23]. While these cross-sectional studies are important in that they establish the initial support of an association between nutrition and mental health, they do not entail causality, which hinders the ability to develop effective interventions to promote healthy eating or mental well-being [24].

With a high prevalence of depressive symptoms in women [25] and among older adults [26], research has attempted to identify protective factors of depression. For instance, consuming an optimal amount of fruit and vegetables [25] or modifying dietary patterns has been suggested to be a promising way to prevent or to reduce depressive disorders [27]. Furthermore, while inconsistent findings have been found across different studies [28–30], cross-sectional studies [31, 32] have found support for the “jolly fat hypothesis”, which postulates that being overweight is associated with lower risk for depression in older adults. Nevertheless, without a clear conceptual understanding of the longitudinal relationship between nutrition and mental health, it is difficult to disentangle whether certain eating habits, such as having a healthy dietary pattern, may influence depressive symptoms over time. The objective of the present study was to fill in a gap in the literature by examining how healthy dietary pattern, BMI, and other covariates (e.g., age, gender, education level, chronic diseases, as well as distance to supermarket and fast food) influence the trajectory of change in depression symptoms among community-dwelling older adults from 2014 to 2017.

## Methods

### Participants

The study used data from a longitudinal study on aging-in-place conducted from 2014 to 2017 [33]. Participants were recruited via invitation letters and phone calls. A total of 2081 older adults aged 65 years and above were recruited from 12 public rental estates for low-income residents in Hong Kong using age-stratification random sampling. Participants were excluded if they had a clinical diagnosis (or history) of schizophrenia, dementia, or intellectual disability. In addition, participants who were currently receiving treatment for depression or bipolar disorder were excluded.

### Ethical approval

The study was approved by the Human Research Ethics Committee of the University of Hong Kong (Reference Number: EA050814 & EA1610004). All participants provided written informed consent.

### Measures

#### Depressive symptoms

Depressive symptoms were assessed using the 15-item Geriatric Depression Scale (validated Chinese version, 34). For each question (e.g., “Do you often feel helpless?”), participants responded *yes* or *no*. A total score is calculated by summing the items that indicate signs of depression, with higher scores indicating higher levels of depressive symptoms. In the current sample, the internal consistency of the GDS was good (α = 0.84).

#### Healthy dietary pattern

Healthy dietary pattern was assessed by an item from the Healthy Ageing Quiz [35]. Participants self-reported the frequency (*rarely/never*, *sometimes*, *most of the time*) of having a balanced diet in a typical week.

#### Body mass index

**Fig. 1:**
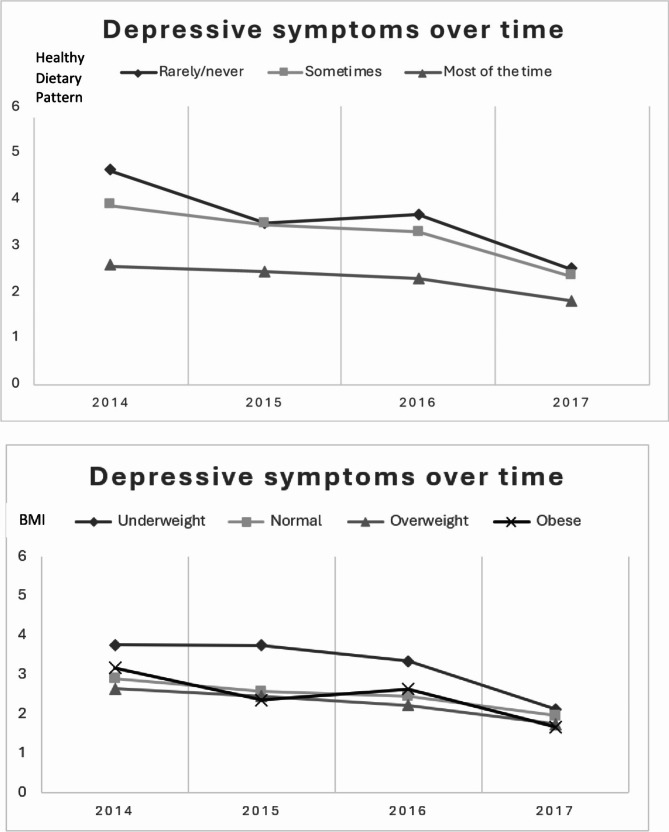
Growth trajectories for depressive symptoms based on diet and BMI

Participants’ height and weight were measured during home visits. Body mass index (BMI) was calculated using the formula kg/m^2^. According to the Centers for Disease Control and Prevention (CDC, 36), BMI can be classified into four broad categories: underweight (< 18.5), healthy (18.50-24.99), overweight (≥ 25), and obesity (≥ 30). While the BMI cut-off is widely used for adults above 20 years old, emerging studies suggest that the current classification may not be appropriate for older adults [37, 38]. More specifically, a higher BMI may have a protective effect on comorbidity [39] and mortality [40]. Thus, BMI was analysed as a continuous variable in the current study. The classification of BMI in Table 1; Fig. 1 is for illustration purposes only.

### Environmental variables

Participants’ residential address was geocoded using spatial buffering analysis [33]. The distance to supermarkets and fast food was calculated from the average central point of each estate, while accounting for the effect of real-world geographical features such as mountains and slopes.

### Statistical analysis

Means and standard deviations were first computed to understand the trajectory of depressive symptoms based on healthy dietary pattern and BMI. Then, growth curve analysis was conducted to examine the impact of a healthy dietary pattern and BMI on the 4-year trajectory of depressive symptoms. First, an unconditional growth model was performed to understand the trajectory of depressive symptoms over time. Then, a conditional growth model was performed to assess the effects of a healthy dietary pattern and BMI on depressive symptoms over time. Age, gender, education level, the number of chronic diseases, and distance to supermarkets and fast food (from residence) were included as covariates. Model fit was examined based on the following criteria: χ^2^/df ≤ 5.0 [41], root-mean-square error of approximation (RMSEA) ≤ 0.06, standardized root-mean-square residual (SRMR) ≤ 0.08, comparative fit index (CFI) ≥ 0.09, and Tucker-Lewis index (TLI) ≥ 0.09 [42]. Data from all participants, including those with incomplete follow ups (e.g., deceased), were included in the analysis. Given the focus of the present study was to examine individual differences in the trajectory of change in depressive symptoms over time, initial levels of depressive symptoms were not being adjusted in the model (a model that adjusts for baseline depressive symptoms is provided in Supplementary Materials). All statistical analyses were conducted using R version 4.2.2. Growth curve analysis was conducted using the lavaan package.

## Results

### Descriptive statistics

Participant characteristics and descriptive statistics are provided in Tables 1 and 2. At baseline, participants had a mean age of 79.65 years (*SD* = 7.94), with a slightly higher percentage of women (56%) than men (44%). Most participants were either married (59%) or widowed (38%), and on average received less than five years of education (*SD* = 4.10). More than half of participants (65%) reported having two or more chronic diseases. Across different rental estates, the distance to supermarket was under 300 m. Overall, the majority of participants reported having a healthy dietary pattern (82%) and fell under the healthy to overweight BMI category (84%).

**Table 1:** Participant characteristics

	M	SD	*n*	%
Age (range = 65–101)	79.65	7.94		
65–74 years			586	28
75-84 years			749	36
>85 years			746	36
Gender				
Male			919	44
Female			1162	56
Marital status				
Single			32	2
Married			1234	59
Separated			6	< 1
Divorced			21	1
Widowed			780	38
Missing			8	< 1
Years of education (range = 0–20)	4.26	4.10		
No formal education			995	48
Primary school			636	31
Secondary school			243	12
High school			163	8
Diploma			23	1
University and above			18	1
Missing			1	< 1
Number of chronic diseases (range = 0–12)	2.41	1.85		
None			283	14
One			452	22
Two or more			1345	65
Missing			1	< 1
Distance (meters)				
Supermarket (range = 126.30-295.25)	211.29	50.4		
Fast food (range = 108.04 to 1015.74)	340.86	296.05		
Healthy dietary pattern	1.79	0.50		
Rarely/never			87	4
Sometimes			271	13
Most of the time			1714	82
Missing			9	< 1
Body Mass Index (range = 12.25–42.54)	23.67	3.96		
Underweight (< 18.5)			175	8
Healthy (18.50-24.99)			1171	56
Overweight (≥ 25)			578	28
Obese (≥ 30)			126	6
Missing			31	2

Note. *M* = mean, *SD* = standard deviation

**Table 2:** Descriptive statistics for depressive symptoms between 2014 to 2017

	Depressive Symptoms M (SD)			
	2014	2015	2016	2017
**All participants**	2.93 (3.34)	2.62 (3.25)	2.48 (3.34)	1.89 (2.90)
**Healthy Dietary Pattern**				
Rarely/never	4.90 (3.92)	3.48 (3.42)	3.66 (3.99)	2.48 (3.19)
Sometimes	3.95 (3.90)	3.45 (3.74)	3.30 (4.01)	2.33 (3.19)
Most of the time	2.67 (3.14)	2.45 (3.13)	2.30 (3.15)	1.80 (2.84)
**Body Mass Index**				
Underweight (< 18.5)	3.76 (3.65)	3.74 (4.04)	3.34 (4.22)	2.12 (3.03)
Normal (18.50-24.99)	2.90 (3.32)	2.57 (3.22)	2.45 (3.26)	1.96 (3.02)
Overweight (≥ 25)	2.64 (3.14)	2.44 (3.05)	2.21 (3.06)	1.74 (2.60)
Obese (≥ 30)	3.16 (3.70)	2.35 (2.91)	2.63 (3.51)	1.67 (2.71)

Note. *M* = mean, *SD* = standard deviation

### Growth curve models

#### Unconditional latent growth curve model

**Fig. 2:**
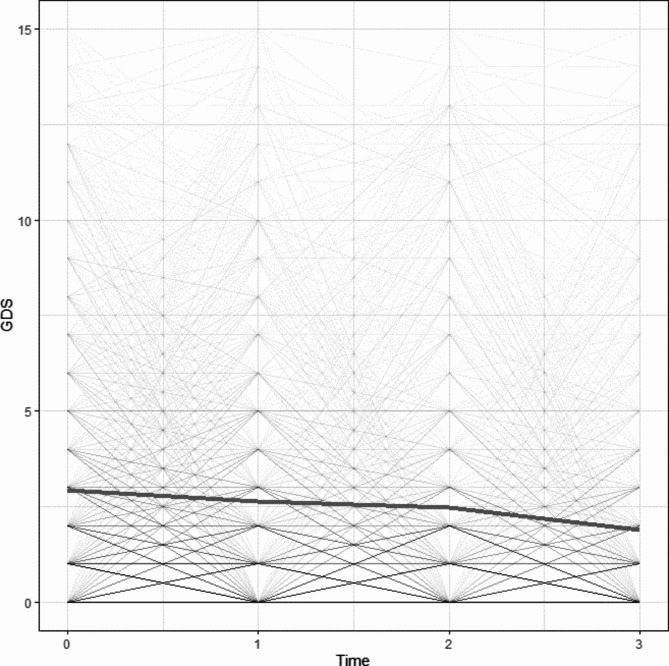
Individual trajectories of depressive symptoms over time. GDS = Geriatric Depression Scale

An unconditional latent growth curve model was first conducted to understand the trajectory of change in depression over time (see Fig. 2). The model demonstrated good fit (χ^2^/*df* = 2.74, RMSEA = 0.04, SRMR = 0.05, CFI = 0.98, TLI = 0.99). Overall, older adults experienced a decrease in depressive symptoms over time (intercept = 2.68, *p* < .001; slope = -0.25, *p* < .001). There was a significant covariance between the intercept and slope (B = -0.39, *p* = .004), indicating that older adults who report higher levels of depressive symptoms at baseline tended to report a steeper decline in depressive symptoms over time. Furthermore, older adults showed significant variability in the initial status of depression (B = 4.88, *p* < .001) and rate of change (B = 0.14, *p* = .03) over time. Given the presence of individual differences in the trajectory of depressive symptoms, we examined whether adding eating habits (i.e., a healthy dietary pattern) and BMI could explain this variation.

#### Conditional latent growth curve model

**Fig. 3:**
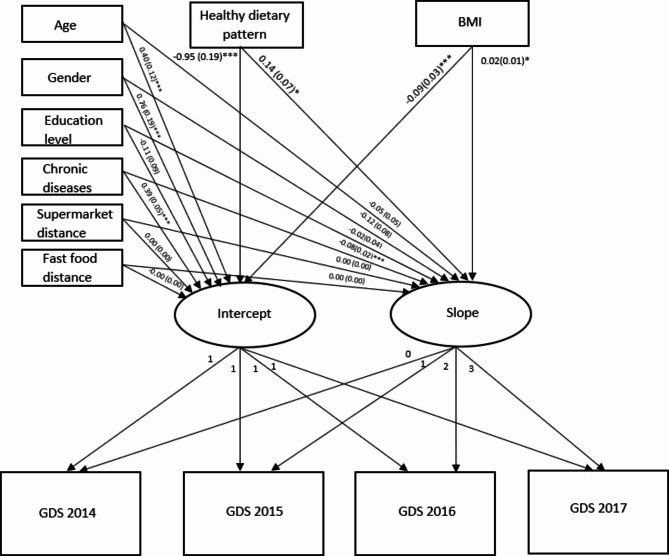
Graphical representation of the conditional latent growth curve model. Unstandardized estimates are shown for each path, with standard errors in parenthesis. BMI = body mass index; GDS = Geriatric Depression Scale. *p < .05, **p < .01, ***p < .001

A conditional latent growth curve model was conducted (see Fig. 3), with dietary pattern and BMI included as time-invariant predictors, while adjusting for age, gender, education level, the number of chronic diseases, as well as distance to supermarkets and fast food from residence. The conditional latent growth model demonstrated a good fit for depression (χ^2^/*df* = 1.76, RMSEA = 0.03, SRMR = 0.02, CFI = 0.98, TLI = 0.97). Consistent with the unconditional latent growth curve model, we found a trend of reductions in depressive symptoms over time (intercept = 4.43, *p* < .001; slope = -0.74, *p* = .04). The non-significant covariance between the intercept and slope (B = -0.22, *p* = .08) indicates that older adults’ initial depressive symptoms were not significantly associated with the rate of change in depressive symptoms over time. Furthermore, while older adults reported significant variability in the initial status of depression (B = 3.79, *p* < .001), the change rate of depressive symptoms (B = 0.12, *p* = .08) was not significantly different.

In examination of the predictors and covariates, we found that healthy dietary pattern, BMI, and the number of chronic diseases significantly predicted the initial status of depressive symptoms (B = -0.95, *p* < .001; B = -0.09, *p* < .001; B = 0.39, *p* < .001, respectively) as well as the rate of change in depressive symptoms over time (B = 0.14, *p* = .05; B = 0.02, *p* = .04; B = -0.08, *p* < .001, respectively). Specifically, older adults with higher diet quality, higher BMI (see Fig. 1), and fewer chronic diseases tended to have lower levels of depressive symptoms at baseline and experience a more gradual (i.e., slower) decline in depressive symptoms over time. While being older (B = 0.40, *p* = .001) or a woman (B = 0.76, *p* < .001) was also associated with higher initial levels of depression, it did not predict the rate of change in depressive symptoms over time (B = -0.05, *p* = .27; B = -0.12, *p* = .12, respectively). As for other covariates we examined, education level, as well as distance to supermarket and fast food did not significantly predict the initial status of depressive symptoms (B = -0.11, *p* = .23; B = -0.00, *p* = .49; B = -0.00, *p* = .20, respectively) or the rate of change in depressive symptoms over time (B = -0.02, *p* = .65; B = 0.00, *p* = .57; B = 0.00, *p* = .20, respectively).

## Discussion

This study examined the trajectory of change in depressive symptoms in older adults. Results indicated that depressive symptoms generally decrease with age, which is supported by previous research that has found a significant decline in the prevalence of depression and a reduction in the severity of depressive symptoms in community-dwelling older adults [43, 44]. Nevertheless, as highlighted by Fiske et al. [45], the impact of depression on older adults should not be overlooked given that there is a higher association between suicidal behaviour and depression compared with other age groups [46]. Thus, identifying potential protective factors for depressive symptoms is vital.

The results suggest that older adults with higher diet quality and higher BMI had lower levels of depressive symptoms at baseline. This is supported by a large body of cross-sectional studies demonstrating a negative association between diet quality and depression in older adults [47–50], as well as depression and BMI [51, 52]. The present study goes beyond the existing literature by demonstrating that higher diet quality and higher BMI (while controlling for covariates) predicted slower declines in depression symptoms over time, compared with those who had lower diet quality and BMI to begin with. While this finding may seem counterintuitive, we postulate that this may be due to the low levels of depressive symptoms reported at baseline (GDS_*M*_ = 2.93 in the current sample, which is lower than GDS_*M*_ = 4.38 in a comparable sample of Chinese community-dwelling older adults recruited between 2014 and 2017, 53), thus there may be less room for further reduction. However, in a further analysis that adjusted for the effects of baseline depressive symptoms in the model (see Supplementary Materials), we found that healthy dietary pattern no longer played a significant role in predicting the decreasing rate of depressive symptoms over time. Future research should replicate these findings with a larger sample size to ensure sufficient statistical power.

Overall, our findings demonstrate that older adults with higher diet quality and higher BMI reported consistently lower levels of depressive symptoms over the 4-year period, as compared to those with poor diet quality and lower BMI. Although not assessed in the current study, it is possible that a higher BMI may protect against muscle loss [54], which is a fundamental contributor to falls, sarcopenia, physical disability, and decreased quality of life [55]. Taken together, this highlights the importance of having a healthy dietary pattern and maintaining a higher BMI, as they may serve as protective factors for depressive symptoms in older adults. In examination of the covariates we included, education level as well as distance to supermarkets and fast food were not significantly associated with depressive symptoms. However, being older, women, and having a greater number of chronic diseases were significantly associated with higher levels of depressive symptoms at baseline. The findings are consistent with previous research suggesting that older women tend to report greater depressive symptoms than men of the same age [56, 57]. Moreover, the positive association found between chronic disease and depression is supported by previous studies which found that older adults with multiple chronic conditions are at higher of risk of depression [58]. We contributed to the literature by demonstrating that while age, gender, and chronic diseases were cross-sectionally associated with depressive symptoms, only chronic diseases had an influence on the rate of change in depressive symptoms over time. Overall, our findings highlight the importance of targeting older adults, women, and those with greater chronic diseases for early depression detection and prevention.

The results of the study provide important implications to target dietary interventions to promote healthy aging. Recent research suggests that dietary interventions have a small positive effect on improving depressive symptoms in clinical and nonclinical adults [59]. Furthermore, research has demonstrated the potential of dietary interventions in preventing and treating depression among individuals who are resistant to seeking mental health support [60]. Provided that mental health stigma is positively associated with age in the Chinese population [61], future research could examine whether dietary interventions represent a more acceptable way to promote mental health among older adults.

A key strength of the current study is that it represents the first to examine the effects of a healthy dietary pattern and BMI on the long-term trajectory of depressive symptoms in a large cohort of community-dwelling older adults. In addition to simultaneously examining the effects of healthy dietary pattern and BMI on depressive symptoms, the present study also accounted for the effects of important covariates, including proximity to supermarkets and fast food, which could have an influence on dietary patterns [62, 63]. Notwithstanding these strengths, there are a few limitations that need to be addressed. First, the current sample represents a low-income and nonclinical sample with low levels of depressive symptoms. Thus, the findings may not be generalisable to older adults with higher income or those with subclinical or clinical depression. Second, healthy dietary pattern was based on self-reported ratings, which may be subject to bias (e.g., underreporting, recall bias) or improper nutrition knowledge [64]. Third, we did not have information on the distance to wet markets, which tends to be more preferred among older adults in Hong Kong [65]. Finally, the present study only followed participants for four years. The limited time-frame may be insufficient to capture the full trajectory of depressive symptoms. Future research should seek to replicate the current findings in different populations, incorporate more objective diet evaluations, and consider longer follow-ups.

## Conclusions

In a sample of older adults residing in Hong Kong, we found that higher diet quality and higher BMI were associated with lower levels of depression over time. While the rate of change in reduction in depressive symptoms was slower for older adults with higher diet quality and higher BMI, the findings nevertheless highlight the importance of maintaining a healthy dietary pattern and higher BMI as they may have protective effects against depression.

## Electronic supplementary material

Below is the link to the electronic supplementary material.

Supplementary Material 1

## Data Availability

No datasets were generated or analysed during the current study.
