# Supplementary material for: The effect of diet quality and body mass index on depression in older adults: a growth curve analysis
Source: BMC Geriatr. 2024 Oct 15;24:834. doi: 10.1186/s12877-024-05392-5 (PMC11476494; doi:10.1186/s12877-024-05392-5)
Supplement: Supplementary file 1 — Supplementary Material 1 [file 12877_2024_5392_MOESM1_ESM.docx]

**The effect of diet quality and body mass index on depression in older adults: A growth curve analysis**

Supplementary Materials

**Conditional latent growth curve model adjusting for baseline depressive symptoms**

A conditional latent growth curve model was conducted with eating habits and BMI included as time-invariant predictors, while adjusting for baseline depressive symptoms, age, gender, education level, the number of chronic diseases, as well as distance to supermarkets and fast food from residence. The model demonstrated a good fit for depression (χ^2^/*df* = 1.72, RMSEA = .03, SRMR = .02, CFI = 0.98, TLI = 0.97), and there was a trend of reductions in depressive symptoms over time (intercept = 4.43, *p* < .001; slope = -0.60, *p* = .09). While older adults reported significant variability in the initial status of depression (B = 4.67, *p* < .001), the change rate of depressive symptoms (B = 0.02, *p* = .87) and the covariance between the intercept and slope (B = -0.01, *p* = .98) were not statistically significant.

In examination of the predictors and covariates, we found that BMI and the number of chronic diseases significantly predicted the initial status of depressive symptoms (B = -0.09, *p* < .001; B = 0.39, *p* < .001, respectively) as well as the rate of change in depressive symptoms over time (B = 0.02, *p* = .05; B = -0.07, *p* = .001, respectively). Although having a healthy dietary pattern (B = -0.95, *p* < .001), being older (B = 0.40, *p* = .001) or a woman (B = 0.76, *p* < .001) was also associated with higher initial levels of depressive symptoms, it did not significantly predict the rate of change in depressive symptoms over time (B = 0.12, *p* = .12; B = -0.04, *p* = .37; B = -0.10, *p* = .19, respectively). As for other covariates we examined, education level, as well as distance to supermarket and fast food did not significantly predict the initial status of depressive symptoms (B = -0.11, *p* = .23; B = -0.00, *p* = .49; B = -0.00, *p* = .20, respectively) or the rate of change in depressive symptoms over time (B = -0.02, *p* = .56; B = 0.00, *p* = .52; B = 0.00, *p* = .24, respectively). Finally, baseline depressive symptoms did not significantly predict the rate of change in depressive symptoms over time (B = -0.03, *p* = .12).

**Figure S1**

Graphical representation of the conditional latent growth curve model. Unstandardized estimates are shown for each path, with standard errors in parenthesis.

BMI = body mass index; GDS = Geriatric Depression Scale.

**p* < .05, ***p* < .01, ****p* < .001
